# Milk Whey Hydrolysates as High Value-Added Natural Polymers: Functional Properties and Applications

**DOI:** 10.3390/polym14061258

**Published:** 2022-03-21

**Authors:** Arely León-López, Xóchitl Alejandra Pérez-Marroquín, Ana Guadalupe Estrada-Fernández, Gieraldin Campos-Lozada, Alejandro Morales-Peñaloza, Rafael G. Campos-Montiel, Gabriel Aguirre-Álvarez

**Affiliations:** 1 Instituto de Ciencias Agropecuarias, Universidad Autónoma del Estado de Hidalgo, Av. Universidad Km 1, Tulancingo C.P. 43600, Hidalgo, Mexico; arely_leon@uaeh.edu.mx (A.L.-L.); pe409780@uaeh.edu.mx (X.A.P.-M.); ca409778@uaeh.edu.mx (G.C.-L.); rcampos@uaeh.edu.mx (R.G.C.-M.); 2 Instituto Tecnológico Superior del Oriente del Estado de Hidalgo, Carretera Apan-Tepeapulco Km 3.5, Colonia Las Peñitas, Apan C.P. 43900, Hidalgo, Mexico; aestrada@itesa.edu.mx; 3 Escuela Superior de Apan, Universidad Autónoma del Estado de Hidalgo, Carretera Apan-Calpulalpan s/n, Colonia Chimalpa Tlalayote, Apan C.P. 43920, Hidalgo, Mexico; amorales@uaeh.edu.mx; 4 Uni-Collagen S.A. de C.V., Arnulfo González No. 203, El Paraíso, Tulancingo C.P. 43684, Hidalgo, Mexico

**Keywords:** milk whey, hydrolysates, immunity, antiviral, antihypertensive, natural polymer

## Abstract

There are two types of milk whey obtained from cheese manufacture: sweet and acid. It retains around 55% of the nutrients of the milk. Milk whey is considered as a waste, creating a critical pollution problem, because 9 L of whey are produced from every 10 L of milk. Some treatments such as hydrolysis by chemical, fermentation process, enzymatic action, and green technologies (ultrasound and thermal treatment) are successful in obtaining peptides from protein whey. Milk whey peptides possess excellent functional properties such as antihypertensive, antiviral, anticancer, immunity, and antioxidant, with benefits in the cardiovascular, digestive, endocrine, immune, and nervous system. This review presents an update of the applications of milk whey hydrolysates as a high value-added peptide based on their functional properties.

## 1. Introduction

One of the most debated topics in food processing is the recycling of the by-products and their applications as a high value-added product. Milk whey represents a clear example of a by-product obtained from cheese production. This material can be considered as a contaminant and at the same time, the source of protein hydrolysates. Whey is a yellowish to greenish clear liquid obtained after milk coagulation during the cheese-making process. Whey represents about 85–95% of the volume of milk volume and contains over 55% of milk nutrients such as minerals, proteins, and lactose [1,2]. Sweet and acid whey are obtained when the coagulation of milk is carried out by enzymatic action (rennet) or the addition of acids posteriorly [3,4]. The most abundant nutrients in whey are: lactose, soluble proteins, lipids, and mineral salts (see Table 1). With the additional presence of some neutral salts such as NaCl, KCl, and calcium salts (primarily phosphate), among others. Aside from these nutrients, whey also contains lactic and citric acids, non-protein nitrogen compounds such as urea and uric acid, and B group vitamins [3,5].

Whey is considered as a waste by-product from the production of cheese. The production of 1 kg of cheese generates approximately 9 kg of whey [7]. It is discarded without treatment to public sewage systems, creating a critical pollution problem. Unfortunately, only 50% of the whey produced globally is used to formulate products. Whey has been traditionally dumped into common water ducts or used to feed livestock. The treatment and re-use of whey is very important as it is one of the most polluting food by/co-product streams; its biochemical oxygen demand (BOD) is around 435,000 ppm and its chemical oxygen demand (COD) is 460,000 ppm [5,8]. Current environmental regulations are forcing cheese makers to treat whey before disposal. The continued growth of the cheese industry, the necessity for reduction in pollutants in the effluent, and the need to maximize returns on raw material have encouraged producers and researchers to seek new ways of using cheese whey with a great amount of research focused on converting this liability into an asset [7,8,9,10,11]. The protein content of whey is one of the main advantages of this by-product. It is known that diet is one of the factors that influence human health and the development of diseases. Proteins are important nutrients in foods that can be hydrolyzed into a wide range of peptides during gastrointestinal digestion. Some of these peptides share characteristics that act in the organism as hormones, neurotransmitters, or regulatory peptides [12]. The importance of whey protein peptides is associated with their functional properties. Some studies have demonstrated the action of these peptides as inhibitors of angiotensin converting enzyme (ACE) on the regulation of blood pressure and enhancement of the immune system. These hydrolysates also help to increase dopamine, improving memory in patients from the geriatric area. In the food industry, whey protein peptides present antimicrobial, antioxidant activities, and also emulsifying properties. All functional properties of whey protein hydrolysates are related to their molecular weight. These properties are in a latent state during the formation of the protein structure complex and is only activated when that structure is broken or hydrolyzed by different methods such as enzymatic action, chemical hydrolysis, and through the application of emerging technologies such as ultrasound and heat treatments [13,14,15,16]. The purpose of this review was to provide an overview of the current understanding of the different methods of extraction of whey protein hydrolysates and the benefits these proteins provide on the body as antiviral, anticancer, antioxidant, and immunological agents. Additionally, an updated overview of their application in different food matrices and improvement in techno-functional properties is described. To accomplish this goal, a scientific literature search was performed through several academic web sites that included Scopus, MDPI, Elsevier, Wiley, SciELO, Web of Science, PubMed, and Redalyc. The topics that we focused on were milk whey classification and composition, milk whey hydrolysates, technologies to obtain these hydrolysates including enzymatic, chemical, and green technologies and included both functional properties of milk whey hydrolysates (antioxidant, antimicrobial, antihypertensive, anticancer, etc.) and applications in food and supplements.

## 2. Intrinsic Properties and Composition of Milk Whey Native Proteins

Whey is regarded as a valuable source of numerous nutritional, functional, and bioactive compounds. Whey presents an elevated content of lactose and proteins that can be used to produce versatile health-oriented compounds [17]; it is also considered a valuable product because of its soluble proteins and high levels of amino acid, vitamins B, lactose, and salt. Whey contains 55–75% and 40–70% of vitamin B6 and vitamin B12, respectively, and also thiamine, nicotinic acid, folic acid and ascorbic acid, riboflavin, and biotin. However, a major concentration of vitamin B12 is displaced in whey during enzymatic treatment compared to acid coagulation [18]. Whey proteins present a high content of essential and branched amino acids such as isoleucine, leucine, and valine. They play an important role as regulators of different metabolic functions, blood glucose homeostasis, and a balanced source of the sulfur-containing amino acids. Minerals such as calcium, magnesium, phosphorus, and trace amount of zinc are present in whey and can act as a base of electrolytes [19,20].

As can be seen in Table 2, whey contains several proteins providing specific functional, physiological, and nutraceutical characteristics, as described below [21,22].

### 2.1. β-Lactoglobulin

β-Lactoglobulin is the main whey protein of the heat coagulable proteins representing approximately 50% of the total protein and approximately 10% of milk protein. Its molecular weight ranges from 8.36 kDa to 18.20 kDa. It occurs as a dimer of two identical subunits consisting of a sulfhydryl group and two disulfide bonds and composed of a 162 amino acid peptide chain. The solubility of this protein depends on pH and ionic strength. Heat denaturation occurs between 70–75 °C [23]. β-Lactoglobulin is not found in breast milk and is considered to be responsible for some allergic reactions in infants fed with cow milk products. For this reason, there are commercial products that imitate human milk based on whey [24,25]. Traditionally, β-lactoglobulin is separated by fractional precipitation with ammonium sulfate at pH with or without heating to cause the precipitation of all serum proteins other than β-lactoglobulin, which are characterized commonly by chromatographic methods such as ion exchange chromatography [26].

Functional activities of whey are related to its composition. Lactose promotes the absorption of magnesium and zinc and is considered better for diarrheal treatment. Additionally, whey proteins show important biological activity and unique functional properties that include high quality nutritional source of amino acids, anti-microbial activity, growth enhancement of beneficial microflora (*Bifidobacteria*), immune-enhancing properties, and the control of specific diseases including cancer and antitoxin activity [1,17,27,28].

The great nutritional value of whey enhances nutraceutical benefits, reducing atherosclerosis, obesity, diabetes, and cancer risk; also, the presence of sulfur amino acids in whey act as cancer prevention agents as forerunners to the strong intracellular cell reinforcement glutathione in one-carbon metabolism. Whey is used as a functional food because it can contribute to the regulation of body weight by providing satiety signals that affect both short-term and long-term food intake regulation [4,27,29,30].

Whey protein has been chosen as an ideal ingredient in diet aiming to prevent or ameliorate metabolic diseases such as obesity because it decreases appetite and increases satiety through several mechanisms such as the regulation of satiety hormones and alteration of hepatic gluconeogenesis [31,32]. Additionally, whey protein is an important component in optimizing body composition because it promotes muscle mass, muscular strength, and muscle hypertrophy in complement with resistance exercises. It induces protein synthesis more efficiently compared to other protein sources due to its faster digestion. This feature leads to a more rapid increase in plasma amino acid levels, particularly in essential amino acids [33,34,35,36]. Furthermore, whey protein subfractions have specific anti-cancer effects because α-lactalbumin and lactoferrin hinder tumor pathways [37]. Whey acts positively in the body by improving the fast absorption of branched chain amino acids. Whey has demonstrated the ability to lower the blood pressure because of an angiotensin-converting enzyme inhibitory property and augmentation of nitric oxide-mediated vasodilation from the component of isoleucine–proline–alanine tripeptide. Furthermore, whey protein consumption can improve lipid metabolism by promoting lipoprotein lipase and inhibiting cholesterol absorption [38,39,40].

### 2.2. α-Lactoalbumin

α-Lactoalbumin represents 11% of total whey proteins. It has a high affinity to calcium and excellent source of essential amino acids mainly represented by tryptophan and cysteine. This protein can be considered as homologous to human α-lactoalbumin because it is 72% analogous in structure. The molecular weight of α-lactoalbumin is around 14 kDa. It has a compact globular structure with four disulfides and denatures at 63 °C, but returns to its natural state on cooling. Whey is an important source of bioactive peptides and essential amino acids including tryptophan, lysine, branched-chain amino acids, and sulfur-containing amino acids. All of them are vital for infant nutrition [41]. It is composed of 123 polypeptides that contain eight cysteine residues. α-lactoalbumin shows some other benefits such as incremental levels of tryptophan in plasma leading to better cognitive performance, good lipid oxidation, better absorption of minerals, antibacterial activity, immunomodulatory effects, and antitumor activity [23,42,43].

### 2.3. Immunoglobulins (Ig)

Immunoglobulins are the largest proteins in milk whey, representing 2% of the total protein in milk. These proteins are composed of three main classes: immunoglobulins IgG, IgA, and IgM. Each form has the same basic structure: two identical light chains of 23 kDa and two chains of 53 kDa. However, IgG is present in a monomeric form, IgA in dimers, and IgM in tetramers. Immunoglobulins are relatively stable to heat and have been incorporated as functional foods because they reduce the risk of gastrointestinal disorder [19,44]. Its main function is to encapsulate bacteria, neutralize toxins, and inactivate viruses. It can also promote gastric digestion, lower blood pressure by reducing cholesterol levels, and it is used in milk formulas for kids as substitutes for milk [23,42].

### 2.4. Bovine Serum Albumin (BSA)

Bovine serum albumin (BSA) represents approximately 5–6% of total milk proteins, and its molecular weight ranges from 66.2 to 66.5 kDa. It is composed of a single polypeptide chain that includes 583 amino acid residues. The cross-linked 17 disulfide bridges of cysteine (Cys) amino acid residues stabilize the structure. Its denaturation temperature is 4 °C. It is also a source of essential amino acids. BSA is able to bind a wide range of ligands including fatty acids, amino acids, drugs, and inorganic ions, and is deemed to be a primary carrier of endogenous and exogenous compounds in the circulatory system [45,46].

The functionality of these proteins has been reported as relevant for their human breast cancer cell inhibitory potential, opioid agonist activity, and antihypertensive property [47,48].

### 2.5. Lactoferrin

This is an iron-binding glycoprotein that belongs to the family of transfer proteins and is generally found in the exocrine secretions of mammalian milk, tears, mucus, and saliva [42,49]. It is a minor component in bovine milk with concentrations of 0.1–0.2 g/L, and has a molecular weight of 80 kDa with a high isoelectric point around 9.5–10. It is composed of a unique polypeptide chain of 700 amino acids; this chain can contain one or two carbohydrate chains. This protein consists of a single polypeptide chain arranged in two highly homologous lobes linked by an a-helix structure. Each lobe contains a ferric iron-binding site. It has 16 intramolecular disulfide bonds but not a free sulfhydryl group [19]. Lactoferrin molecules are thermostable and resistant to acids at pH 4; they are also resistant to the action of trypsin and chymotrypsin but can be hydrolyzed with pepsin. Its ability to bind iron generates various biological functions such as the inhibition of bacteria and fungi growth, promoter of certain cell lines, prevention of lipid peroxidation, and good absorption of iron in the body. Its applications include health supplements, functional foods and beverages, infant formulas, cosmetics, and oral care products [47,50].

### 2.6. Lactoperoxidase

This is a glycoprotein present in the mammary, salivary, and lacrimal glands of mammals with a molecular weight of 78 kDa. This enzyme is a unique polypeptide chain with 612 amino acids. Lactoperoxidase is relativity stable to heat, it resists pasteurization treatment (72 °C, 15 s), and it can be inactivated at 78 °C. This glycoprotein can catalyze the oxidations of several substrates including fatty acids, aromatic amines, phenols, and aromatic acids [51]. Lactoperoxidase plays an important role in the protection of the lactating mammary gland and the intestinal tract of neonates against pathogenic microorganisms; it can also be used in combination with other materials for the production of films for food packaging. It is also involved in the degradation of certain carcinogens and in the protection of animal cells against peroxidative effects. All these functional properties allow this enzyme to be used in the food, cosmetics, pharmaceutical, and agricultural industries [52,53].

### 2.7. Protease–Peptone

This is defined as a heterogeneous mixture of whey proteins; it is thermostable and soluble at acid pH values. It can be separated by heat treatment and adjustment of the pH to 4.6. Proteose peptone 3 (PP3) represents the major factor of proteose peptone, it is a phosphorylated glycoprotein with low- molecular-weight fraction and surface-active property [47,54]. There is great interest in the food industry for protease–peptone because it has shown a good emulsifying activity in the oil-in-water emulsion model used in products with soya bean oil and ice cream. It presents excellent foam-forming properties [55,56] and is the fraction of the milk that remains soluble when the milk is heated at 95 °C for 20 min under acidic conditions. This protein acts as an immunomodulator, anti-bacterial, and also inhibits the activity of lipase [23,57].

### 2.8. Glycomacropeptide

Glycomacropeptide (GMP) is a peptide found in cheese whey, separated by the action of enzymatic action (rennin) on κ-casein proteins. It is the glycolyzed form of the casein macro peptide. The glycolmacropeptide is a soluble peptide of 64 amino acids with a molecular weight of 6.8 kDa. It contains variable amounts of oligosaccharides, mainly galactosamine, galactose, and sialic acid, is available as an ingredient for its application in food, beverages, cosmetics, functional and medicinal supplements, and is also associated with biological benefits and anti-infective and antioxidant activities [58,59].

Food supplementation with GMP exerts several health potentials because it acts as an immunomodulator and anti-inflammatory protein. It has a prebiotic effect on *Bifidobacterium* and *Lactobacillus* sp. It also enhances calcium absorption, improving bone health and inhibits the adhesion of several cariogenic bacteria including *Sobrinus*, *Sanguis,* and *Streptococcus mutans* [19,47,54].

## 3. Hydrolyzed Protein from Milk Whey as High Value-Added Compounds

The valorization of a waste product can be defined as a process that transforms waste through physical, thermal, chemical, or biological methods in order to create products that can be incorporated as part of the circular economy into production chains. Whey valorization focuses mainly on the concentration and transformation of lactose, proteins, or any other nutrients into new value-added compounds [60,61]. Some value-added compounds from whey have been extracted from different biotechnological approaches such as enzymatic, microbial, thermal, galacto-oligosaccharide probiotics (GOS), lactose fatty acid esters, biocolorants, aroma compounds, and bacterial cellulose [62].

Galacto-oligosaccharides (GOS) are a well-known class of probiotics or substrates that are selectively utilized by host microorganisms, conferring a health benefit [63]. GOS have various benefits to human health including the selective stimulation of the beneficial intestinal bacteria growth, maintenance of the normal flora balance in the intestine, increased calcium absorption, and decreased serum cholesterol levels and cancer risks. The health-promoting effects of GOS include immunomodulation, lipid metabolism, mineral absorption, weight management, and obesity-related issues, among others [64,65,66].

Lactose fatty acid esters are odorless, non-toxic, and biodegradable compounds of high importance for the food, cosmetics, and pharmaceutical industries. Lactose fatty acid esters have been recognized for their superior properties as attractive substitutes of synthetic surfactants, excellent emulsifying and stability properties in food products. Additionally, they present antimicrobial activity against many foodborne pathogens as well as medicinal properties such as anticancer activity [67,68,69]. 

Carotenoids are one of the most important natural pigments and can usually be extracted from plants. However, cheese whey, or deproteinized cheese whey, has been used for the production of carotenoids by using various microorganisms (*Blakeslea trispora*, *Mucor azygosporus*, *Rhodotorula rubra*) for the fermentation of various carbon sources such as glucose, sucrose, and xylose. Carotenoids possess biological functions such as antioxidant activity, reduction in cardiovascular diseases, anti-diabetic, anti-cancer, and anti-inflammation activities. The interest in the carotenoids from whey focuses on the use of low-cost substrates to reduce the production costs [70,71,72]. Fermentation is also an alternative way for the production of natural aroma compounds from milk whey and involves the use of several yeast strains such as *Metschnikowia pulcherrima*, *Bacillus licheniformis*, *Wickerhamomyces pijperi*, and *Saccharomyces cerevisiae* [73,74,75,76]. 

Bacterial cellulose (BC) is a biopolymer with important physiochemical properties such as water holding capacity, hydrophilicity, high degree of polymerization, mechanical strength, crystallinity, and porosity. All these BC characteristics represent a wide range of potential applications starting from the food industry and biomedicine to electronics and cosmetics. Bacterial cellulose extracted from whey through enzymatic and acidic pre-treatments can be considered as a cheaper growth medium for BC production due to the low-cost of raw materials as well as its enhanced BC yields [77], reducing environmental pollution from dairy waste. BC has been used as an edible antimicrobial food coating increasing shelf life as well as a healthy food supplement for patients with gastrointestinal disorders, obesity, cardiovascular diseases, and diabetes. BC is considered as a multifunctional food ingredient because it can be used to improve the rheology of foods as a fat replacer ingredient for the production of both low-calorie and low cholesterol food products [78,79]. 

## 4. Methods of Extraction of Whey Hydrolysates

**Figure 1:**
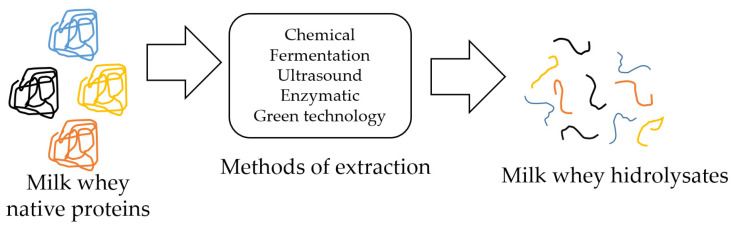
Different extraction methods of milk whey hydrolysates.

Milk proteins have been considered as the most important source of bioactive peptides; after their ingestion, these peptides can positively influence the cardiovascular, digestive, endocrine, immune, and nervous systems. Peptides represent a functional food because they not only satisfy the nutritional needs, but also help to reduce the risk of health problems [80]. Whey represents 95% of milk weight so it is a good source of bioactive peptides that can be produced by hydrolysis by applying different methods: enzymatic action, chemical treatment (acid or alkaline), microbial fermentation with proteolytic bacteria, ultrasound, thermal process, and others (Figure 1) [81,82].

Hydrolysis of proteins by chemical processes using an alkaline or acidic media commonly using NaOH, KaOH, HCl at different concentrations is more difficult to control and generates hydrolysates with modified amino acids. Table 3 shows that chemical treatment presents several and important disadvantages: reduces nutritional quality, oxidizes cysteine and methionine, destroys some serine and threonine, and the conversion of glutamine and asparagine to glutamate and aspartate, respectively [83,84,85].

Fermentation of native whey proteins produces peptides or free amino acids valuable for their functional properties [86]. Many lactic acid bacteria (LAB) such as *Lactococcus lactis*, *Lactobacillus helveticus*, *Lactobacillus delbrueckii* ssp. *Bulgaricus*, *Bacillus* spp., and *Bifidobacterium* have proteolytic action in whey [87]. Fermentation has an advantage; hydrolysis is carried out by proteases of microorganisms, and thus, bioactive peptides can be purified without further hydrolysis. However, during fermentation, some of the peptides and/or amino acids released from the native proteins are used as a substrate for strain growth [17,83,88]. Another treatment to obtain hydrolysates from whey is high-energy power ultrasound. This method has been used successfully as it improves enzymatic hydrolysis, producing bioactive peptides. Ultrasound (>20 kHz) generates high temperature and pressure, causing physical and chemical changes at the molecular levels and consequently, better access of enzymes to hydrolysis sites [89,90]. The ultrasound method induces the unfolding of whey protein by high cavitation (20 kHz). This method also changes the secondary structure of proteins, decreasing the content of α-helices and increasing β-sheets and β-turn. Ultrasound treatment improves functional properties such as in vitro angiotensin converting enzyme inhibitor (ACE) and immunomodulatory activities [89,91,92]. The most common hydrolysis treatment is enzymatic; the functionality of these hydrolysates depends on different factors such as the type of enzyme, pH, temperature, time, and enzyme/substrate ratio [93,94]. Compared to chemical hydrolysis, enzymatic hydrolysis usually takes place under relatively mild operating conditions (temperature 20–70 °C, pH 6.0–8.0) [95]. The most widely used enzymes to produce whey hydrolysates are proteases and are capable of promoting specific and selective protein modifications. Trypsin is a commercial enzyme widely used for protein hydrolysis. This enzyme is highly active, has elevated cleavage specificity, and is very stable under different experimental conditions [93,96,97].

Not only are animal source enzymes used to obtain whey hydrolysates, some enzymes from plant sources such as papain have also been used [98,99,100,101]. Additionally, plant crude extracts were used for the hydrolysis of whey proteins, some examples are described as follows: extracts from Citrus aurantium flowers, trompillo (*Solanum elaeagnifolium*) berries, and melon (*Cucumis melo*) fruit [83,102]. However, enzymatic hydrolysis can modify the nutritional value of the hydrolysates and other properties such as solubility, emulsification, foaming, and gelation and bitter products [103].

Emerging technologies such as thermal treatments (>90°C), high hydrostatic pressure (100–1000 MPa), and even ultrasound can modify the characteristics of hydrolysates, creating a large number of hydrophobic groups, increasing antioxidant, ACE inhibitory, and immunomodulatory activities and also maintaining the original sensorial quality and nutrients [92,104,105].

These methods are environmentally-friendly (no generation of chemical waste) and are very promising because they increase the amount of whey hydrolysates, functional properties, and reduced time of hydrolysis [96,106,107,108,109].

**Table 3:** Advantages of different methods of the extraction of whey hydrolysates.

Methods of Extraction	General Characteristics	Advantages	References
Chemical	Difficult to control and generates hydrolysates with modified amino acids.	Easy access to reagents.	[83,84,85]
Fermentation	It involves some acid lactic bacteria (BAL), no need to use acid or alkaline media	Bioactive peptides obtained can be purified without further hydrolysis.	[17,88]
Ultrasound	>20 kHz induced the unfolding of whey protein by high cavitation	Improves the enzymatic hydrolysis producing bioactive peptides from proteins presents in whey.	[89,90,91,92]
Enzymatic	Takes place under relatively mild operating conditions	Not addition of chemical reagents, nutritional value is maintained, control of the process (time, temperature and pH), most common method.	[93,94,95,110]
Green technology	Can be thermal treatments and high hydrostatic	Reduce time of hydrolysis, no generation of chemical waste.	[96,106,107,109]

## 5. Functional Properties of Hydrolyzed Milk Whey Proteins

**Figure 2:**
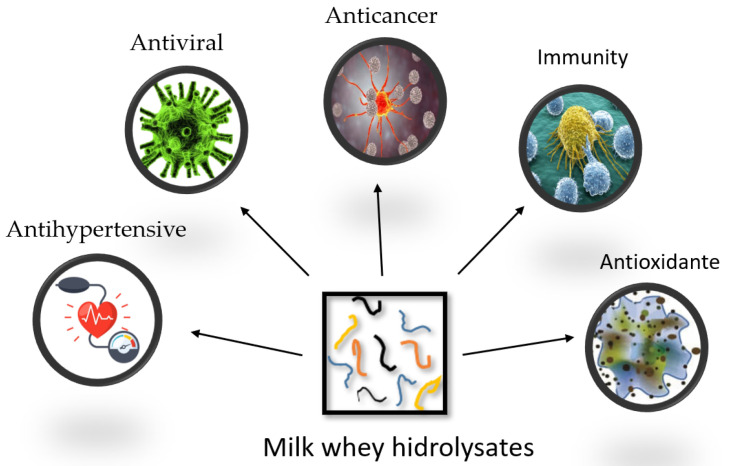
Functional properties of milk whey hydrolysates.

Milk whey biological functions are mainly related to the cardiovascular, digestive, endocrine, immune, and nervous systems. However, many of the bioactive peptides are encrypted in native whey protein, so in order to liberate these peptides, it is necessary to apply hydrolysis methods that generate milk whey hydrolysates. In recent years, milk whey hydrolysates have been studied due to their potential as a functional ingredient capable of producing beneficial effects on health such as immunity, antioxidant, anticancer, antiviral, and antihypertensive (Figure 2). At the same time, production of hydrolysates can be an interesting approach in adding value to whey protein, while at the same time protecting the environment from their pollutant effects [82,83,93,111].

### 5.1. Antihypertensive

Management of hypertension is a multifactorial issue and must be accompanied by different prevention-oriented activities such as ACE-inhibitory drugs prescription, lifestyle changes including weight loss, quitting smoking, and reducing sodium and alcohol intake. Aside from these recommendations, milk-derived peptides obtained by fermentation have shown excellent ACE-inhibitory capacity, and thus a blood pressure-lowering effect [112].

Cardiovascular diseases are the main cause of death around the world. The renin–angiotensin system is the pathway that exerts control over blood pressure. The angiotensin converting enzyme (ACE) is responsible for altering blood pressure in the body. ACE is responsible for converting angiotensin I into angiotensin II, providing a vasoconstrictor effect [113,114]. The use of synthetic drugs to control these diseases causes several side effects such as cough, taste disturbance, and skin rash, among others. Therefore, an alternative for the prevention and/or treatment of arterial hypertension is the use of bioactive components obtained from natural sources (animal or vegetable) such as antihypertensive peptides [115,116,117].

Several studies have demonstrated the mechanism of action of peptides for ACE inhibition [118]. It can be described in two different points. First, by physiologic importance: oral administration of peptides reaches the bloodstream in an active form to exert their antihypertensive effect, since gastrointestinal digestion and transport are the main barriers to the bioavailability of peptides. Second, the digestion of peptides via gastrointestinal proteases could be used as a process for the production of peptides with ACE inhibitory capacity [116].

Miralles and co-workers [119] reported advances in the field of study of antihypertensive peptides. They concluded that food derived antihypertensive peptides represent a good source of functional agents in healthy diets. However, after oral intake, these peptides are hydrolyzed during digestion and absorption, rendering shorter peptide forms that have been revealed to exert a potent and more sustained antihypertensive effect. This peptide biotransformation is the reason why the technique in in vitro ACE-inhibitory activity is not sufficient to demonstrate their antihypertensive effect. Studies on whey peptides oriented to inhibit ACE activity are very scarce; this might be because the structure of β-lactoglobulin is resistant to digestive enzymes [120].

It is well-known that casein-derived tripeptides such as valyl-prolyl-proline (Val-Pro-Pro) and isoleucyl-prolyl-proline (Ile-Pro-Pro) present excellent antihypertensive properties as shown in vivo [121,122]. Additional benefits have also been reported such as when oral intakes of these bioactive tripeptides including fermented milk and casein hydrolysates attenuated atherosclerosis development in apolipoprotein E-deficient mice.

### 5.2. Antiviral

Peptides are considered as ideal alternatives for synthetic therapeutic agents. The mechanism of peptide action depends on their structure and can be enhanced by modification of the native form. The antiviral action of peptides can be generated through three mechanisms: (I) peptides that inhibit virus adhesion and cell membrane fusion; (II) peptides that disrupt viral envelope; and (III) peptides that inhibit virus replication by interacting with viral polymerase. Lactoferrin and its hydrolysates have antiviral multiactivity against virus-like adenovirus, poliovirus, rotavirus, hepatitis B virus, Zika, dengue virus, influenza virus A H1N1, and respiratory syncytial virus. The antiviral action depends on the time of incubation with the virus, the concentration of the hydrolysates, and the method of obtention. Hydrolysates, with a molecular weight under 10 kDa composed of basic, aliphatic and polar amino acids with an isoelectric point greater than 10, present excellent antiviral effect due to their capacity to form amphipathic structures. Their good efficacy, safe, selectivity, and predictable metabolism are the main strengths of peptides in drug production [123,124,125,126,127].

### 5.3. Anticancer

Food-derived protein hydrolysates or isolated peptides possess anticancer activities through various molecular mechanisms. This includes the stimulation of apoptosis, arrest of cell cycle profession, cell membrane damage, inhibition of cell adhesion, topoisomerases, modulation of immune response, and inhibition of intracellular signaling [128,129]. It has been reported that some predominant hydrophobic amino acids of peptides such as proline, leucine, glycine, alanine, and one or more residues of lysine, arginine, serine, threonine, and tyrosine play an essential role in anticancer activities [130]. The anticancer activity of peptides is based on their structural characteristics such as amino acid composition, sequence, and hydrophobicity. The lower the molecular weight of peptides, the greater the molecular mobility and diffusivity for interactions with cancer cell components and thus stronger anticancer activity. Peptides obtained from lactoferrin decreased metastasis and a significant delay in growing tumors [128,131,132].

### 5.4. Immunity

The mechanism of immunomodulatory activity occurs mainly through the activation of macrophages, stimulation of phagocytosis, increased leukocyte count, increased induction of immune modulators such as cytosines, immunoglobulins, stimulations of NK cells, stimulation effect on splenocytes, and activation of mitogen-activated protein kinase. This mechanism depends on the amino acid sequence, composition, length, and structure that peptides can modulate immune responses [128].

α-Lactoalbumin is a small protein composed of 123 amino acids and a molecular weight around 14 kDa [5,133]. Hydrolyzation of this protein provides an antihypertensive effect [134] as well as antimicrobial [135] and immunostimulatory properties [136]. Proteins function as antigens that present regions called epitopes, which are identified by antibodies and subsequently trigger an allergenic reaction [137,138,139].

Isothiocynates have been linked to health properties and result from the degradation of glucosinolates, which is found in plants such as cauliflower, broccoli, and cabbage [140,141,142].

Spötell et al. [143] carried out immunological staining in native untreated and benzyl isothiocyanate (BITC)-modified α-lactoalbumin performed directly on the plate after their separation using high performance chromatography-immunostaining (HPTLC-IS) analysis. They reported that the HPTLC immune staining procedure did not destroy the tertiary and secondary structure of the protein. The chemical modification of protein with BITC derived from structural changes of the protein molecule and influenced the increase in allergenicity.

### 5.5. Antioxidant

The antioxidant activity of peptide fragments has been investigated in different vegetable sources such as soy bean [144] and pea seed [145]. In beverages, the combination of nutritional properties of milk whey and banana passionfruit were reported to increase the antioxidant properties of the beverage due to the presence of phenolic compounds. The higher the content of pulp, the higher the antioxidant activity [146]. Additionally, healthy functional beverages based on whey milk added with soursop [147] and raspberry were reported to increase their antioxidant properties as well as antihypertensive activity [148].

## 6. Applications of Milk Whey Proteins Hydrolysates

Whey hydrolysates are commonly applied to a wide range of food applications (dairies, bakeries, meet products, beverages, food supplements or functional foods) due to their nutritional validity, functional activities, and cost effectiveness. Whey hydrolysates are also used to replace other proteins, improving the functional properties of many food products. Whey protein hydrolysates are important in food processing due to their technological properties including oil and water holding, emulsifying capacity, foam capacity, and solubility. They can promote the formation of volatile compounds in food products regardless of whether they are added in small quantities [149,150,151,152].

Whey-based beverages such as dairy, not dairy, fermented, or non-fermented show functional activity because of their highly nutritional and digestible properties associated with the presence of hydrolysates as well as their functional properties such as antioxidant, antimicrobial, antihypertensive, and others (see Table 4) [153,154].

Mann et al. [154] prepared a flavored milk beverage with the addition of whey hydrolysates and good antioxidant activity attributed to the existence of several peptides contained in it. Additionally, Arranz et al. [155] developed a whey protein-based beverage with the same characteristics and no effect on apparent viscosity and stability of the beverage. Ferreira et al. [148] prepared a whey-raspberry flavored beverage that presented antioxidant capacity and ACE inhibition. Some no dairy beverages have been developed with hydrolysates that very often contain citrus fruits (mainly orange, followed by lemon, rarely grapefruit) as well as mango, passionfruit, pear, apple, and strawberry. The addition of milk whey hydrolysates in these types of beverages increased sensory and physicochemical properties such as flavor, odor, low sedimentation, and storage stability [156,157,158]. The addition of milk whey hydrolysates with antioxidant and antimicrobial activities into a beverage appears to create an exciting link between food science and therapeutic nutrition [153]. However, the use of high amounts of hydrolysate could result in negative effects in appearance and aroma [152].

Several researchers have investigated the application of milk whey hydrolysates as a food supplement. These studies have demonstrated that consumption of whey hydrolysates and other sources of protein hydrolysates such as soy, casein, and wheat presented high protein synthesis in the body [159,160,161]. Fassina et al. [162] demonstrated that milk whey hydrolysates are an excellent source of nutritious and commercially available alternative food sources commonly used as a food supplement by athletes. This supplement provides them with essential amino acids and bioactive peptides. Lockwood et al. [163] concluded that whey protein supplementation increased muscle mass after eight weeks in college-aged males. Hansen and co-workers [164] demonstrated that consumption of whey protein hydrolysates before an exercise session, followed by ingestion of more protein hydrolysates plus carbohydrates for a training period of six weeks, improved specific mitochondrial protein adaptations compared to the intake of carbohydrates. Additionally, milk whey hydrolysate supplementation showed increments in muscle mass and strength over a 10-week experiment in older post-menopausal women [165]. Brown and colleagues [166] reported that milk whey supplementation improved the recovery of muscle function and flexibility, accelerating the repair of damaged skeletal muscle and thus its force generation capacity. The consumption of this type of supplementation may contribute to reduced immunosuppression and excessive inflammation, accelerating muscle function recovery after heavy training [162,167].

**Table 4:** Applications of MWH and functionality.

Product	Functionality	Reference
Flavored milk beverage	Antioxidant activity	[154]
Whey MWH food supplementation in post-menopausal women	Increase muscle mass and strength	[165]
Apple juice	Low sedimentation	[156]
Beverage enriched white flaxseed oil	Increased of flavor, odor	[157]
MWH food supplementation in college-aged males	Increase mixed muscle and protein synthesis	[163]
MWH food supplementation	Improved recovery of muscle function and flexibility	[166]
Whey-raspberry flavored beverage	Antioxidant capacity and ACE inhibition	[148]
MWH food supplementation in athletes	Excellent source of nutritious	[162]
Whey protein-based beverage	Antioxidant and antimicrobial activity, no affecting physicochemical properties	[155]
Protein supplementation	Increasing mixed muscle and protein synthesis and lean body mass	[164]

## 7. Future Considerations of Milk Whey Hydrolysates

The numerous treatments applied to whey proteins offer an opportunity for future researchers to modify their textural and structural properties, improving the functionality and obtention of low molecular weight hydrolysates. This includes enzymatic treatments, emerging technologies such as ultrasound, high pressure, and thermal processes. Although milk whey hydrolysates have been applied to a wide variety of food products oriented to human health, most of the reported experimental data have been documented through a series of studies based on in vitro models as well as animal systems. Scientists face challenges in the near future in the implementation of clinical trials in humans. Additionally, the commercialization of these natural polymers must comply with the health regulations of different regions. The claims related to health effects should be supported by scientific studies. Finally, the use of new enzyme selections with known specificity could offer new functionality and applications to hydrolyzed whey proteins, for example, in the fields of antigenic response, health maintenance, and healing.

## 8. Conclusions

Although considered as a waste product, the literature supports that milk whey has relevant nutritional and functional properties that make it suitable for use in the food industry. Whey presents an important content of proteins, group B vitamins, minerals, and lactose. These proteins can be hydrolyzed by different methods (enzymatic, with LAB, ultrasound, thermal process, and others) obtaining low molecular weight peptides. Several studies have shown that its consumption as a food supplement helps protein synthesis in the body and increases muscle mass. Furthermore, milk whey protein in its hydrolyzed form possesses functional properties such as antioxidants, antihypertensive, anticancer, antivirals, and immunomodulatory activity. The molecular weight and properties of whey hydrolysates depend on the hydrolysis method and can be used in different industries including functional foods. The applications of milk whey are not limited to the food industry; this review confirmed the wide range of uses and advantages of milk whey hydrolysates. Future investigations must be conducted under scientific methods, oriented toward human trials for the elucidation of their benefits on the human system.

## Figures and Tables

**Table 1:** Comparison of sweet and acid whey components [6].

Characteristics	Sweet Whey	Acid Whey
pH	>5.6	<5.6
Water	93–94%	94–95%
Protein (g/L)	6–10	6–8
Lactose (g/L)	46–52	44–46
Minerals (g/L)	2.5–4.7	4.3–7.2
Obtained by	Enzymatic action	Organic acids

**Table 2:** Protein composition of whey [21,22].

Protein	Content (g/L)
β-lactoglobulin	2.9
α-lactoalbumin	0.6
Inmunoglobulin	0.3
Serum albumin	0.6
Lactoferrin	0.1
Lactoperoxidase	0.03
Protease-peptone	1
Glycomacropeptide (GMP)	0.9
